# SS-31, a Mitochondria-Targeting Peptide, Ameliorates Kidney Disease

**DOI:** 10.1155/2022/1295509

**Published:** 2022-06-06

**Authors:** Yuexin Zhu, Manyu Luo, Xue Bai, Jicui Li, Ping Nie, Bing Li, Ping Luo

**Affiliations:** Department of Nephrology, The Second Hospital of Jilin University, Changchun, Jilin 130041, China

## Abstract

Mitochondria are essential for eukaryotic cell activity and function, and their dysfunction is associated with the development and progression of renal diseases. In recent years, there has been a rapid development in mitochondria-targeting pharmacological strategies as mitochondrial biogenesis, morphology, and function, as well as dynamic changes in mitochondria, have been studied in disease states. Mitochondria-targeting drugs include nicotinamide mononucleotide, which supplements the NAD+ pool; mitochondria-targeted protective compounds, such as MitoQ; the antioxidant coenzyme, Q10; and cyclosporin A, an inhibitor of the mitochondrial permeability transition pore. However, traditional drugs targeting mitochondria have limited clinical applications due to their inability to be effectively absorbed by mitochondria *in vivo* and their high toxicity. Recently, SS-31, a mitochondria-targeting antioxidant, has received significant research attention as it decreases mitochondrial reactive oxygen species production and prevents mitochondrial depolarization, mitochondrial permeability transition pore formation, and Ca^2+^-induced mitochondrial swelling, and has no effects on normal mitochondria. At present, few studies have evaluated the effects of SS-31 against renal diseases, and the mechanism underlying its action is unclear. In this review, we first discuss the pharmacokinetics of SS-31 and the possible mechanisms underlying its protective effects against renal diseases. Then, we analyze its renal disease-improving effects in various experimental models, including animal and cell models, and summarize the clinical evidence of its benefits in renal disease treatment. Finally, the potential mechanism underlying the action of SS-31 against renal diseases is explored to lay a foundation for future preclinical studies and for the evaluation of its clinical applications.

## 1. Introduction

The kidneys are important metabolic and endocrine organs, and kidney disease is a global health concern that has resulted in high economic costs worldwide [1]. The kidneys, specifically proximal tubules, have a high mitochondria density due to the large amount of ATP required for solute reabsorption, blood waste product removal, and fluid and electrolyte balance regulation [2]. Mitochondria coordinate the tricarboxylic acid cycle and produce ATP through oxidative phosphorylation while releasing reactive oxygen species (ROS) [3]. ROS, as important upstream inducers of kinases and epigenetic factors, play a key role in cell signaling [4]. At low levels, ROS are important intracellular and intercellular signals necessary for maintaining kidney homeostasis and function; however, high ROS levels disrupt cellular balance and mediate oxidative stress damage, thereby causing apoptosis, inflammation, and fibrosis [5]. ROS are key factors involved in the development and progression of various renal diseases, including ischemia-reperfusion (IR) injury, drug-induced acute kidney injury (AKI), chronic kidney disease (CKD), diabetic nephropathy (DN), hypertensive kidney damage, and other glomerular diseases [6–10]. Considering that mitochondrial dysfunction leads to a decrease in ATP supply and excessive ROS production, which in turn triggers cellular damage, oxidative stress, apoptosis, inflammation, and fibrotic responses, moreover, renal diseases can also affect mitochondrial function through multiple pathways, including mitochondrial bioenergetics, membrane integrity, calcium homeostasis, and mitochondrial dynamics [11]. Therefore, for kidney disease treatment, protecting mitochondria may be more effective than targeting individual downstream events.

SS peptides are novel mitochondria-targeting antioxidants centered on alternating aromatic residues and basic amino acids, which were discovered fortuitously by Szeto and Schiller while carrying out studies on opioid receptors [12]. Among them, D-Arg-Dmt-Lys-Phe-NH2 (SS-31, also known as MTP-131, elamipretide, and Bendavia, collectively known as SS-31) was first reported in the early twenty-first century and has been extensively studied [13]. SS-31 has dimethyl tyrosine residues that interact with oxygen radicals forming unreactive tyrosine radicals. The tyrosine radicals couple together to form di-tyrosine, enabling it to scavenge oxygen radicals and inhibit linoleic acid and low-density lipoprotein oxidation. Further, SS-31 accumulates on the inner mitochondrial membrane, protects and restores mitochondrial structure, promotes ATP synthesis, reduces electron leakage and cardiolipin peroxidation, and has no effect on healthy mitochondria [14–16]. Thus, SS-31 exhibits protective effects against various diseases, including cardiac, neurological, respiratory, retinal, kidney, and aging-related diseases, as well as sepsis and diabetes [17–23]. However, to date, studies on SS-31 in renal diseases are limited, and its action mechanism remains explored. Moreover, a comprehensive summary and discussion of the existing studies are lacking. Considering this, this article reviews the pharmacokinetics of SS-31, its possible molecular mechanisms in the treatment of renal diseases, and relevant preclinical and clinical studies on SS-31, and finally discusses its potential targets in the treatment of renal diseases, with the aim developing a new management path in the renal field.

## 2. Pharmacokinetics and Safety of SS-31

SS-31 is a cell-permeable aromatic cationic tetrapeptide with a molecular weight of 639.8 g/mol which consists of a series of small water-soluble peptides [24]. At physiological pH, SS-31 carries a net charge of 3+ and selectively targets and accumulates in the inner mitochondrial membrane via electrostatic and hydrophobic interactions. Studies carried out using isolated mitochondria have revealed that SS-31 is concentrated approximately 5000-fold in the mitochondria [25].

SS-31 is small, easy to synthesize, soluble in water, not easily degraded by peptidase, and very stable in solution. It is not dependent on mitochondrial energy and membrane potential and is absorbed by cells in an unsaturated manner in the absence of receptor- or transporter protein-mediated processes. In addition to cellular uptake, it can also be transported across cells; it is thus rapidly absorbed after administration, with peak plasma levels detectable within 15 min and attaining a steady state within 30 min. It can be distributed to the kidneys, heart, liver, lungs, and skeletal muscle, with its highest concentrations observed in the kidneys [26, 27]. SS-31 is completely excreted by the kidneys, and 100% of the peptides and peptide metabolites are detected in the urine. Pharmacokinetic studies have shown that it is not only rapidly distributed in vivo, but has a long half-life, with an elimination half-life of approximately 2 h in rats, dogs, and monkeys [28].

SS-31 accumulates primarily in the inner mitochondrial membrane and is not transported into the mitochondrial matrix, even at very high concentrations; it has no effects on normal mitochondria and is therefore considered relatively safe. In clinical studies, the main adverse effects associated with SS-31 were found to be injection site adverse reactions, such as erythema (57%), pruritus (47%), pain (20%), urticaria (20%), and irritation (10%), most of which were mild. In addition to injection site adverse reactions, dizziness and headaches were also observed. No serious adverse reactions or fatalities were observed [29–32]. In an atherosclerotic renal artery stenosis phase IIa clinical trial, all patients tolerated a single SS-31 infusion without developing adverse clinical effects such as fever, headaches, vomiting, hematuria, or allergic reactions. There was no change in serum creatinine (Scr) levels and urine cytology 24 h after SS-31 infusion [33]. These results demonstrate the safety and tolerability of therapeutic SS-31 and provide a basis for its widespread application. However, large-scale studies are required for validation.

## 3. Mechanism of Action of SS-31 against Kidney Disease

**Figure 1:**
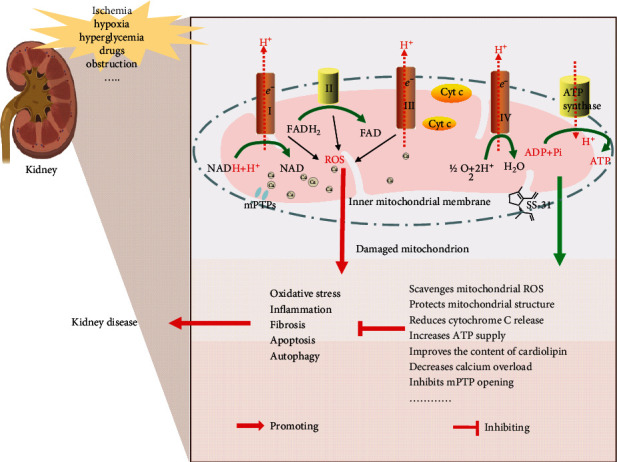
Mechanism of action of SS-31 against kidney disease. Mitochondria produce ATP and ROS through an electron transport chain consisting of complexes I to V. Ischemia, hypoxia, hyperglycemia, drugs, obstruction, genetic, and other risk factors can affect the kidneys, causing mitochondrial dysfunction and kidney disease. SS-31 protects mitochondrial structure, scavenges ROS, increases ATP supply, reduces cytochrome C release, and inhibits mPTP opening and calcium overload by binding to cardiolipin in the inner membrane of mitochondria, thereby exerting anti-oxidative stress, anti-inflammatory, antifibrotic, antiapoptotic, and autophagic effects.

As a mitochondria-targeting drug, SS-31 exerts significant effects by the binding of cardiolipin, promotes electron transfer, inhibits cytochrome C peroxidase activity, and reduces electron leakage. Although the action mechanism of SS-31 against kidney disease is not fully understood, the available studies suggest that it elicits its effects primarily by protecting mitochondrial structure, repairing damaged mitochondria, scavenging ROS, and increasing ATP supply, thereby reducing oxidative stress and improving apoptosis, inflammatory response, autophagy, and fibrosis [14, 27, 34] (Figure 1).

ROS damage kidney cells by oxidizing membrane phospholipids, proteins, nucleic acids, and carbohydrates [35]. Mitochondria are both organelles that produce ROS and the main sites for ROS action. The antioxidative stress effect of SS-31 has been demonstrated in a model of IR kidney injury and in kidney disease caused by various pathological conditions [14, 36]. In a cisplatin-induced AKI model, SS-31 was found to reduce mitochondrial and intracellular ROS levels, inhibit the expression of downstream NLRP3 vesicles, and improve renal oxidative stress and apoptosis [37]. In addition, SS-31 was shown to inhibit NADPH oxidase activity in a contrast-induced AKI model, thereby ameliorating acute tubular necrosis [38]. SS-31 significantly inhibited Nox4 expression and NADPH oxidase activity in a diabetic kidney model and in renal mesangial cells cultured in a high-glucose environment; it also suppressed thioredoxin-interacting protein expression and reduced ROS production and oxidative stress [15]. Furthermore, SS-31 was found to prevent podocyte and renal tissue damage in diabetes patients by scavenging for mitochondrial ROS (mtROS) and breaking the vicious oxidative stress cycle [39]. Moreover, in type 2 diabetes patients, SS-31 reduced ROS production and regulated endoplasmic reticulum stress and autophagy, as well as intracellular environmental homeostasis [40, 41]. CD36, a transmembrane protein, can reportedly induce ROS production, and SS-31 downregulates CD36 expression and regulates ROS production [42]. During unilateral ureteral obstruction (UUO), mechanical traction, oxidative stress, and ischemia cause apoptosis, which leads to macrophage infiltration and interstitial fibrosis. SS-31 may alleviate apoptosis, epithelial mesenchymal transition, and fibrosis by scavenging for ROS, thereby ameliorating renal injury [43, 44].

In addition to scavenging for ROS, SS-31 optimizes electron transport and ATP synthesis, inhibits lipid peroxidation, prevents the opening of the mitochondrial permeability transition pores (mPTPs), inhibits mitochondrial swelling, and reduces cytochrome C release and calcium overload [45]. In IR injury, ATP depletion due to ischemia leads to AKI, and timely recovery of ATP during reperfusion is essential to reverse renal injury. However, IR causes mPTP opening in the inner mitochondrial membrane, leading to mitochondrial swelling and depolarization, as well as delayed ATP recovery [46]. In contrast, SS-31 inhibited mPTP during early reperfusion, accelerated ATP supply, protected mitochondrial structure and respiration, and reduced apoptosis and necrosis of renal tubular epithelial cells [14]. SS-31 inhibits cardiolipin oxidation by interacting with cardiolipin on the inner mitochondrial membrane, protecting the mitochondrial cristae membrane, and restoring ATP supply [27].

Mitochondrial morphology and function are inextricably linked, and SS-31 protects the morphology of mitochondria. Obesity causes mitochondrial shrinkage and structural disorganization in proximal tubules and podocytes of the kidney, reduction of cristae, and rupture of the outer mitochondrial membrane, and SS-31 lengthens mitochondria and restores cristae membrane structure [23]. Cardiolipin is essential for maintaining the normal structure of the inner mitochondrial membrane, and insufficient synthesis of this molecule can lead to various mitochondria-related diseases [47]. The ability of SS-31 to restore cardiolipin levels, thereby improving associated renal damage, has also been demonstrated in studies on post-ischemic CKD and renal artery stenosis, among others [48–50]. In addition, ATP-binding cassette A1 (ABCA1) deficiency leads cardiolipin accumulation and mitochondrial dysfunction, thereby inducing podocyte damage and DN progression. SS-31 inhibits cardiolipin peroxidation, participates in central renal phospholipid remodeling, and regulates the fusion between immature or mature long-chain cardiolipin and mitochondria, thus providing a novel therapeutic strategy for DN [51, 52]. Notably, SS-31 not only protects mitochondria but also restores mitochondria damaged in disease states. The progressive loss of mitochondrial structure and function with increasing age leads to age-related glomerulosclerosis. A previous study revealed that SS-31 could repair age-related mitochondrial morphology abnormalities and glomerular sclerosis [53]. The disruption of the mitochondrial structure of podocytes after ischemia can also be reversed by SS-31 [48].

Interestingly, in addition to directly regulating oxidative stress, promoting ATP synthesis, and protecting mitochondrial structures, SS-31 was found to act on the renin-angiotensin system (RAS). In acute tubular and glomerular injury models, it was found to be involved in the regulation of Ang receptors (ATR) and aminopeptidase A, thereby limiting renal damage by downregulating AT1R expression, inhibiting aminopeptidase A activity, and upregulating protective AT2R mRNA levels [54]. This finding suggests that SS-31, as well as other AT2R modulators, may be potential target drugs for kidney disease treatment.

## 4. Preclinical Evaluation of the Effects of SS-31 against Kidney Disease

### 4.1. SS-31 in Animal Experiments

The protective effects of SS-31 have been shown in various renal disease models, including IR injury-induced AKI, drug-mediated AKI, CKD, DN, and glomerular and renal vascular-related disease models (Table 1).

#### 4.1.1. SS-31 and AKI

Using an IR kidney injury model, Szeto et al. found that SS-31 reduced Scr levels, increased creatinine clearance, reduced tubular necrosis and apoptosis, and preserved brush border structure in a group of experimental rats at both 30 and 45 min following bilateral renal blood flow blockade and that it showed a significant dose-dependent effect after 45 min of ischemia. Electron microscopic observation of mitochondrial structures after 45 min of ischemia demonstrated that SS-31 treatment preserved the integrity of mitochondrial cristae, increased the number of viable mitochondria, and promoted the recovery of ATP [14]. In another study, Liu et al. further evaluated the long-term effects of treatment with SS-31 on peritubular and glomerular capillaries after 4 weeks of ischemia. They found that SS-31 protected endothelial cell mitochondria and inhibited TNF-*α* expression and lymphocyte and macrophage infiltration, thereby inhibiting TGF-*β*-induced interstitial fibrosis. However, continuous SS-31 administration for 4 weeks showed no additional benefits when compared with a single SS-31 injection before the induction of ischemia [55]. To verify whether mitochondrial damage persists in the long term after ischemia and to explore the role of SS-31 in preventing AKI toward CKD, Szeto et al. evaluated rats subjected to 45 min of bilateral renal ischemia for 9 months and observed significant mitochondrial damage in their endothelial cells, podocytes, and proximal tubular cells. However, 6 weeks of SS-31 administration after 1 month of induced ischemia was found to repair the damaged mitochondria, prevent glomerulosclerosis and fibrosis, and normalize upregulated IL-1*β* and IL-18 levels; these effects were sustained for over 6 months following treatment [48]. The present results suggest that long-term administration of SS-31 after AKI is effective and provides durable protection after the termination of drug therapy.

Hypercholesterolemic rats were administered diatrizoate meglumine for AKI induction, and early intraperitoneal SS-31 injection reduced Scr levels and increased creatinine excretion. Moreover, the diatrizoate-induced decrease in SOD and ATP levels and increase in NADPH, MDA, and Nox4 expression levels in renal tissues were reversed by SS-31 [38]. In cisplatin-induced AKI, SS-31 may exert antioxidative stress and antiapoptotic effects by modulating mtROS production, downregulating the NLRP3-IL-1*β*/caspase 1 signaling pathway, ameliorating cisplatin-induced acute tubular necrosis, and reducing Scr and blood urea nitrogen (BUN), but not body weight, renal weight, serum albumin, and triglycerides [37]. Cecal ligation and puncture caused sepsis in mice, and intraperitoneal administration of 5 mg/kg of SS-31 restored renal ATP supply, reduced apoptosis and renal tissue damage, and improved Scr and BUN levels [56, 57].

To test the protective effect of SS-31 in acute glomerular and tubular injury, Jean et al. administered aristolochic acid and driamycin to BALB/c mice to induce acute tubular interstitial injury and glomerular injury, respectively. In both models, SS-31 exhibited promising renoprotective effects. However, the modulatory effects of SS-31 on the indicators of inflammation, oxidative stress, and cell proliferation were model-related. Among them, SS-31 treatment did not reduce the upregulation of aristolochic acid-induced inflammation or downregulate the expression of cell cycle protein-dependent kinase 2, except for the expression of NF-*κ*B, which may be related to the overly severe renal lesions on which aristolochic acid acts. In contrast, SS-31 for driamycin-treated mice yielded good gains in both aspects. However, in addition to the regulation of acute oxidative stress and inflammatory responses, SS-31 is involved in the regulation of the RAS [54].

#### 4.1.2. SS-31 and Atherosclerotic Renal Vascular Disease

Atherosclerotic renal artery stenosis is an important causative factor for cardiovascular disease and promotes CKD progression. Although percutaneous renal angioplasty (PTRA) and stenting can restore blood pressure, they cannot restore deteriorated kidney structure and function [62]. Eirin et al. investigated a swine atherosclerosis model and found that SS-31 infusion 30 min before and 3.5 h after PTRA effectively reduced the adverse effects associated with this technique. By increasing mitochondrial biogenesis and decreasing PTRA-induced inflammation, oxidative stress, apoptosis, and fibrosis, SS-31 improved renal microvascular rarefaction, glomerular filtration rate (GFR), and tubular damage [36]. However, whether mitochondrial dysfunction promotes renal remodeling as well as dysfunction in atherosclerosis has not been elucidated. Afterward, using a swine atherosclerotic nonrevascularization model, they revealed that cardiolipin stabilization, attenuation of cardiolipin pathological remodeling, and mitochondrial protection reduced stenotic renal fibrosis and improved renal and renal vascular function [49]. Using the same model, Kim et al. found that SS-31 ameliorated the renal fibrosis and decrease in renal function associated with atherosclerotic renal stenosis; however, it only partially alleviated atherosclerotic renal artery stenosis-induced renal cellular senescence, a finding which refutes the strong causal relationship that is said to exist between mitochondrial dysfunction and cellular senescence during the early stages of atherosclerotic renal artery stenosis. In addition, whether or not SS-31 affects long-term renal aging needs to be further investigated [50]. In addition to its protection against atherosclerosis-induced renal injury, SS-31 reduced Scr and downregulated TNF-*a* and TGF-*β* expression in a porcine model of coronary artery stenosis but had no effect on renal hemodynamics and endothelial nitric-oxide synthase (eNOS) expression, which contradicts Eirin's findings. Eirin et al. demonstrated that SS-31 inhibits eNOS downregulation during atherosclerotic renal stenosis and in a metabolic syndrome-induced vascular injury model; this inconsistency may be due to the fact that different mechanisms underly renal injury development [49, 58].

#### 4.1.3. SS-31 and UUO

During UUO, mechanical pulling of the tubular epithelium and oxidative stress leads to tubular cell injury and death; in addition, subsequent obstruction-induced kidney ischemia aggravates tubular cell injury. Damaged tubular cells release proinflammatory factors and chemokines, which induce macrophage infiltration and the inflammatory response; these further release profibrotic factors, such as TGF-*β*, which play an important role in UUO-induced fibrosis [63]. In the UUO model, SS-31 (1 or 3 mg/kg) was intraperitoneally administered to rats one day before obstruction was induced and then continuously administered for 14 days. It was found to significantly reduce fibroblast and macrophage infiltration, improve apoptosis and renal tubular cell regeneration, and was more potent at a dose of 3 mg/kg. Further analysis of oxidative damage indicators in the kidney revealed that SS-31 reduced the expression of 8-hydroxy-2′-deoxyguanosine (8-OHdG) and heme oxygenase-1 (HO-1). However, SS-31 attenuated renal fibrosis, but had no effect on TGF-*β* and chemokine receptor-1, which are important indicators of fibrosis [44]. Using the same model, Liu et al. found that obstruction-induced renal fibrosis, oxidative stress, and apoptosis were exacerbated by hypochlorite-modified albumins, suggesting that oxidative stress-induced mitochondrial damage plays an important role in obstructive nephropathy. These effects can be prevented by SS-31 [59].

#### 4.1.4. SS-31 and DN

In unilateral nephrectomized streptozotocin (STZ) diabetic CD-1 mice, Hou et al. found that continuous 8-week intraperitoneal SS-31 injection attenuated proteinuria, glomerular hypertrophy, and renal fibronectin and type IV collagen accumulation through a mechanism that may be related to the inhibition of p38 MAPK activation and the prevention of oxidative stress. However, this study ignored the crucial role of inflammation in DN and did not explore the effect of SS-31 on inflammation [15]. In addition, treatment with SS-31 has been shown to decrease the protein expression levels of Drp1, which responds to mitochondrial fission, and to increase the expression of Mfn1, a mitochondrial fusion protein. These findings suggest that SS-31 ameliorates renal apoptosis, oxidative stress, and fibrosis by regulating mitochondrial dynamics, thereby decreasing proteinuria and Scr and BUN levels, with no effect on blood glucose levels or body weight [60]. Miyamoto revealed that SS-31 protected normal cardiolipin synthesis, and remodeling maintained mitochondrial superoxide levels by improving DN and increased Mfn1 protein expression but had no significant effect on Drp1 protein, which is slightly different from the findings of Yang et al. [52].

CD36 is a class B scavenger receptor that mediates ROS production during DN, participates in oxidative damage during type 2 diabetes, and plays a role in the mechanism of apoptosis [64, 65]. In addition, CD36 is responsible for lipid deposition in several tissues [66]. Hou et al. found that maintaining balance between ROS production and the antioxidant system is one of the mechanisms by which SS-31 exerts its renoprotective effects, and that improving CD36-mediated lipid deposition is also an effective mechanism by which SS-31 improves DN. In addition, SS-31 inhibited the activation of the NF-*κ*B signaling pathway [42]. This study also remedied the missing information from the previous experiments about the interaction between SS-31 and inflammation. ABCA1 is ATP-dependent and regulates cholesterol and phospholipid efflux. The inhibition of ABCA1 expression increases susceptibility to DN as well as DN progression. Ducasa et al. found that ABCA1 deficiency causes mitochondrial dysfunction by decreasing cardiolipin content and impairing its function, thereby predisposing podocytes to damage in the diabetic context. ABCA1 upregulation and the inhibition of cardiolipin peroxidation both significantly impaired DN progression and ameliorated podocyte damage, while SS-31 reduced cardiolipin oxidation, thereby decreasing proteinuria, mesangial expansion, and podocyte deficiency in ABCA1-deficient ob/ob mice [51].

#### 4.1.5. SS-31 and Other Kidney Diseases

After 28 weeks of a high-fat diet in mice exhibiting obesity-associated nephropathy, Szeto et al. found that SS-31 prevented obesity-associated glomerulosclerosis, mesangial expansion, macrophage infiltration, and endothelial cell and podocyte apoptosis but had no effect on body weight, glucose levels, or insulin resistance in mice. This finding suggests that the protective effects of SS-31 are independent of its effects on metabolic indicators; however, it elicits its effects by protecting renal cell mitochondria, restoring renal AMP kinase activity, and preventing intracellular lipid accumulation and endoplasmic reticulum stress [23]. Metabolic syndrome (MetS)-induced mitochondrial structural and functional dysfunction is closely associated with glomerular hyperfiltration, medullary hypoxia, and intrarenal microangiopathy. Eirin et al. demonstrated that SS-31 attenuates necrosis, apoptosis, and tubular damage in the kidneys of pigs with high cholesterol/carbohydrate diet-induced MetS by preventing cardiolipin loss, restoring mitochondrion counts and remodeling, and improving myeloid oxidation [61]. SS-31 was found to improve renal microvascular remodeling, vessel tortuosity, and increased vascular density in the MetS state by protecting mitochondria. Moreover, SS-31 protected the endothelial function of renal arteries because the vasodilatory response to acetylcholine in resected renal arterial rings was impaired in MetS but normal in MetS/SS-31. However, in healthy subjects, SS-31 did not affect renal structure, function, or redox status [67].

Recently, Nastaran et al. evaluated the efficacy and safety of SS-31 against autosomal dominant polycystic kidney disease during pregnancy using pregnant Pkd1RC/RC mice and found that it downregulated ERK1/2 phosphorylation, improved mitochondrial supercomplex formation, and inhibited the progression of autosomal dominant polycystic kidney disease. In addition, they found that SS-31 crossed the placenta and was present in breast milk, and no teratogenic or harmful effects were observed [68].

### 4.2. SS-31 in Cellular Experiments

Several cellular experiments have demonstrated the protective effects of SS-31 against renal diseases (Table 2). Eirin et al. found that treatment of swine renal artery endothelial cells with 30 mM tert-butyl hydroperoxide resulted in reduced cardiolipin content and mitochondrial dysfunction and that co-culture with SS-31 restored normal cardiolipin content and reduced caspase-3 levels and superoxide anion and nitrotyrosine synthesis. This suggests that SS-31-induced cardiolipin content restoration reduces apoptosis and oxidative stress. In addition, SS-31-treated cells exhibited increased eNOS expression, supporting a direct role of mitochondrial protection in improving nitric oxide bioavailability [49].

After treatment of 30 mM glucose-cultured mesangial cells with 100 nM SS-31, SS-31 was found to inhibit high-glucose-induced ROS generation, stabilize mitochondrial membrane potential and ATP levels, inhibit cytochrome C release, and alleviate apoptosis, thereby protecting mesangial cells against damage under high-glucose conditions [15]. The high-glucose-induced generation of ROS, activation of NADPH kinase, and expression of CD36 and NF-*κ*B were inhibited by SS-31 when 100 nM of SS-31 was added to high-glucose-induced HK2 cells and co-cultured for 48 h [42]. Similarly, Yang et al. treated HK2 cells with 5-30 mM glucose and subsequently with 100 nM SS-31 for 72 h; they found that SS-31 reversed mitochondrial swelling and cristal membrane breakage and inhibited ROS production and the upregulation of Drp1 protein expression. Notably, the pretreatment of HK2 cells with Drp1 inhibitor (Mdivi1) under high-glucose conditions resulted in a similar protective effect to SS-31 [60]. In human podocytes, SS-31 inhibited C3a-induced foot cell motility [69]. The cardiolipin-mediated, ABCA1-dependent susceptibility to DN was explored using siABCA1 podocytes cultured from the sera of patients with progressive diabetes. The podocytes were treated with SS-31, and the toxicity of diabetic sera was found to be blocked by SS-31 [51].

## 5. Clinical Trials

Current studies on SS-31 have mostly focused on cardiac disease and mitochondrial myopathy, with fewer clinical studies dedicated to kidney disease. A randomized, double-blinded, placebo-controlled phase IIa preliminary clinical study (NCT01755858) was carried out on 14 patients with severe atherosclerotic renal artery stenosis who required PTRA for severe hypertension and/or decreased renal function. These patients were randomly assigned to the SS-31 group (0.05 mg/kg/h, 6 patients) or the placebo group (8 patients); the drug was intravenously infused into the patients 30 min before PTRA was performed and during PTRA. Subsequently, 24 h after PTRA, partial tissue hypoxia was observed in both groups; however, the degree of hypoxia was significantly lower in the SS-31 group. Moreover, 3 months after PTRA, only patients in the SS-31 group showed increased renal blood flow (202 ± 29 to 262 ± 115 ml/min, *P* = 0.04) and renal cortical perfusion (1.99 ± 0.8 to 2.9 ± 1 ml/min/ml). In addition, there was a decrease in systolic blood pressure and improvements in Scr levels and eGFR in the SS-31 group as compared to the placebo group. SS-31 was well tolerated throughout treatment, and there were no reported adverse effects such as fever, rash, headache, or nausea [33].

Another phase 1 study is registered on the Clinical http://Trials.gov/ website (NCT02436447). It is an open, parallel, multidose study that investigates the mean peak blood concentration of SS-31 in patients with varying renal function after a one-hour intravenous infusion of SS-31 administered for 7 consecutive days. Unfortunately, there have been no published results to discuss.

## 6. Discussion and Future Directions

**Figure 2:**
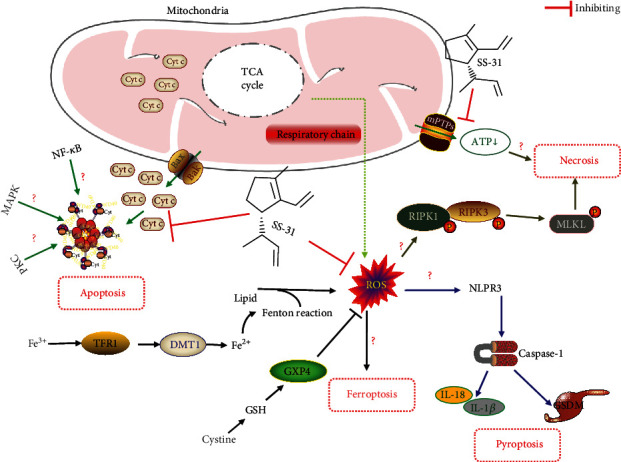
SS-31 potential protective mechanisms and signaling pathways. SS-31 concentrates on the inner mitochondrial membrane, by scavenging ROS, increasing ATP synthesis, and inhibiting mPTP opening; it can reduce lipid peroxidation, thereby improving ferroptosis, apoptosis, necrosis, and pyroptosis. In addition, SS-31 may also regulate apoptosis through PKC, MAPK, and NF-*κ*B signaling pathways.

Cell membranes are particularly susceptible to lipid peroxidation caused by ROS-induced oxidative damage owing to their high polyunsaturated fatty acid content. Lipid peroxidation directly damages phospholipids and induces cell death through apoptosis, necrosis, pyroptosis, or ferroptosis [70]. In addition, mitochondria play an important regulatory role in the death signaling pathway through the release and recruitment of specific death-promoting factors [71]. Therefore, in addition to targeting mitochondria for the regulation of oxidative stress, inflammation, fibrosis, and apoptosis, SS-31 may also exert its protective effects by regulating other death pathways, such as necrosis, pyroptosis, and ferroptosis, as well as other signaling pathways (Figure 2).

Apoptosis is a form of cell death that is essential for maintaining homeostasis in the body. The mitochondrial pathway is an intrinsic apoptotic pathway. The opening of mitochondrial mPTP leads to cytochrome C release into the cytoplasm; cytochrome C, together with apoptosis protease activator 1 and pre-caspase-9, constitutes apoptotic vesicles, which promote apoptosis [72]. Lipid peroxidation can also induce apoptosis by stimulating intra- and extracellular signaling pathways [73]. NF-*κ*B is a key regulatory component of the antiapoptotic signaling pathway [74]. Lipid peroxidation products reportedly inhibit NF-*κ*B transcription factors and IKK, and this in turn induces Bcl-2 downregulation, thereby leading to apoptosis [75]; this suggests that lipid peroxidation can regulate apoptosis through the NF-*κ*B signaling pathway. In addition, lipid peroxidation initiates the apoptotic process by forming a complex with ERK, JNK, and p38 to activate MAPKs and caspase signaling. Lipid peroxidation can also activate the PKC pathway to regulate apoptosis [76]. As discussed above, SS-31 can improve apoptosis by protecting mitochondria, which has been validated in relevant animal models. However, studies on other mechanisms and signaling pathways that can improve apoptosis are lacking. ROS-mediated lipid peroxidation products can regulate apoptosis through the NF-*κ*B, MAPK, and PKC pathways, and SS-31 can inhibit ROS production. Further investigations are required to determine whether SS-31 can target these pathways to improve apoptosis.

Ferroptosis is a novel form of regulated cell death caused by severe lipid peroxidation; it is dependent on ROS production and iron overload. Studies have revealed that ferroptosis is associated with a variety of diseases, such as neurological disorders, cardiomyopathy, cancer, and lung disease [77–80]. Regarding kidney disease, ferroptosis reportedly plays an important role in the development of AKI and DN [81, 82]. ROS accumulation is considered to be a key factor in ferroptosis induction [83]. Mitochondria generate ATP through the citric acid cycle and initiation of electron transport chain activity providing energy to the cell, and this process produces ROS as well as lipid peroxides that induce ferroptosis. Krainz et al. found that nitrogen oxides, such as XJB-5-131 and JP4-039, could prevent ferroptosis in HT-1080, BJeLR, and panc-1 cells [84]. MitoQ, a ubiquinone derivative that targets mitochondria, was found to restore the significant disruption in mitochondrial morphology and function induced by RSL3 (a small molecule that induces ferroptosis) [85]. As a mitochondria-targeting antioxidant, SS-31 can scavenge for ROS, which are involved in the process of ferroptosis; this provides a theoretical basis for the involvement of SS-31 in the regulation of ferroptosis. However, no studies have been carried out in this area in kidney disease; thus, whether SS-31 can alleviate kidney disease by inhibiting ferroptosis has a good prospect for discussion.

pt?>Necrosis is a regulated caspase-independent form of cell death [86]. Necrosis is associated with inflammatory diseases and ischemic injury, making this mode of cell death an important therapeutic target. The absence of the mitochondrial cristal membrane leads to inadequate ATP supply, and upon ATP depletion, the cell undergoes necrosis [87]. In addition, mitochondria-derived ROS promote necrosome initiation by promoting RIPK1 autophosphorylation, which leads to necrosome formation [88]. Cyclophilin D is an important regulatory mPTP component that may participate in necrotic signaling by promoting mPTP opening [89]. Therefore, based on the mechanism of necrosis and the potential role of SS-31, regulating necrosis using SS-31 may also be an effective strategy for the treatment of renal diseases.

Pyroptosis is a lysis-programmed cell death associated with inflammation; it is a key fibrotic mechanism that plays an important role in kidney disease development [90]. MtROS triggers the activation of NLRP3 inflammatory vesicle caspase-1, which cleaves the proinflammatory cytokines, IL-1*β* and IL-18. The activated caspases in turn cleave and activate gasdermin D, which forms pores in the plasma membrane and increases its permeability, thereby leading to pyroptosis [91]. In this context, ROS production may play a key role in the activation of pyroptosis and become an interesting target for the regulation of pyroptosis. Several recent studies have revealed that a number of ROS-scavenging antioxidants, including nuclear factor erythroid 2-related factor 2 (Nrf2) inducers and polyphenols with antioxidant properties, can reduce renal pyroptosis [92–95]. A recent study by Zuo et al. revealed that anesthesia and surgery activate NLRP3 inflammatory vesicle caspase-1-dependent pyroptosis and that SS-31 not only exerts protective effects against mitochondrial dysfunction, but also attenuates surgery-induced pyroptosis [96]. It has also been suggested that SS-31 ameliorates LPS-induced nucleus pulposus cell apoptosis, pyroptosis, and inflammation by scavenging for ROS, maintaining balance in mitochondrial dynamics, and inhibiting NF-*κ*B pathway and NLRP3 inflammasome activation [97]. However, studies on the effects of SS-31 on renal pyroptosis are lacking. Novel strategies for pyroptosis management may be developed when the action mechanism of SS-31 is comprehensively elucidated.

Autophagy can be induced by ROS, endoplasmic reticulum stress, and hypoxia. Mitochondrial autophagy (mitophagy) is a type of autophagy that is used to remove excess or damaged mitochondria [98]. Studies have revealed that abnormal or defective mitophagy is central to the pathophysiology of several kidney diseases and that the activation of mitophagy is a protective mechanism against kidney disease [99]. This shows that pharmacological studies targeting mitophagy to treat kidney disease are promising. Coenzyme Q10, an important component of the mitochondrial electron respiratory chain, also has antioxidant properties. Sun et al. found that the activation of mitophagy through the modulation of the Nrf2/ARE signaling pathway exerted beneficial effects against DN and that the ROS-targeting antioxidant, mitoTEMPO, not only restored mitochondrial mitophagy, but also improved renal function in db/db mice co-treated with ML385 (Nrf2 inhibitor) and coenzyme Q10 [100]. Xiao et al. observed reduced mitophagy in the renal tubules of high-glucose environment and STZ-induced diabetic mice, while the mitochondria-targeted antioxidant MitoQ reversed mitophagy deficiency and ameliorated diabetic kidney injury [101]. However, there is a gap in research on SS-31 and mitophagy in renal diseases, and further in-depth investigations are required.

## 7. Conclusions

Mitochondrial dysfunction is involved in the development and progression of kidney disease, and methods to target mitochondria for disease treatment have been developed. However, there are only a limited number of drugs available whose effectiveness has yet to be evaluated. Presently, the use of mitochondrial antioxidants represents a novel therapeutic approach, and among them, SS-31 shows promising applications. However, only a few preclinical studies have been carried out on the usefulness of SS-31 in the treatment of renal diseases, and only a single model has been used. Thus, it is crucial for diverse renal disease models to be developed for the exploration of the mechanisms and signaling pathways underlying the action of SS-31. Of note, large-scale, multisample, multicenter clinical studies need to be carried out to evaluate the efficacy, safety, and tolerability of SS-31 for better clinical application. In addition to proteomics and genomics, studies on SS-31 have revealed that lipidomics is a potential target for drug research.

## Figures and Tables

**Table 1:** In vivo study of SS-31 in kidney disease.

Disease models	Renal protective effect	Cytokines	Renal function	Renal pathology	References
IR-induced AKI in rat	Mitochondrial protection, anti-inflammatory, antioxidative	↑GSH ↓MDA, HO-1, MPO	↓Scr, BUN	↓Tubular necrosis, tubular cell detachment	[14]
IR-induced AKI in rat	Mitochondrial protection, antifibrotic, anti-inflammatory	↓TNF-*α*, CD68^+^macrophages, CD3^+^ lymphocytes, TGF-*β*, *α*-SMA	↓Scr, BUN	↓Endothelial cell injury, renal microvascular rarefaction	[55]
IR-induced AKI in rat	Mitochondrial protection, anti-inflammatory	↓TNF-*α*, IL-18, IL-1*β*	NA	↓Tubulointerstitial fibrosis, glomerulosclerosis, podocyte swelling	[48]
Contrast-induced AKI in rat	Antioxidative	↓MDA, NADPH, Nox4 ↑SOD, ATPase	↓Scr, FeNa%, FeK% ↑Ccr	↓Vacuolar degeneration, tubular dilation, protein cast, epithelial cell shedding	[38]
Contrast-induced AKI in mice	Antioxidative, antiapoptotic	↓NLRP 3, IL-1*β*, caspase 1, ROS, MDA	↓Scr, BUN	↓Tubular necrosis, tubulointerstitial lesions	[37]
Sepsis-induced AKI in	Mitochondrial protection, antioxidative, antiapoptotic, anti-inflammatory	↓TNF-*α*, MDA, NF-*κ*B, iNOS, ROS, MPO	↓Scr, BUN	↓Kidney injury scores	[56, 57]
Renal artery stenosis in swine	Mitochondrial protection, antioxidative, antiapoptotic, anti-inflammatory	↓Caspase 3, Bcl-2 associated X-protein, PGC1*α*, PPAR-*α*, TNF-*α*, MCP-1, collagen IV↑VEGF	↑GFR	↓Tubular injury score, tubulointerstitial fibrosis, glomerular score ↑microvascular density	[36]
Renal artery stenosis in pig	Mitochondrial protection, antifibrotic	↓Superoxide anion ↑Cardiolipin, COX-4	↑GFR, renal blood flow	↓Glomerulosclerosis, fibrosis	[50]
Coronary artery stenosis in pig	Anti-inflammatory antifibrotic	↓TNF-*α*, P67, GP91, TGF-*β*	↓Scr	↓Renal fibrosis, glomerular score, tubular injury	[58]
UUO in rat	Antioxidative	↓Caspase 3, HO-1, p38 MAPK, NF-*κ*B-p65	NA	↓Interstitial fibrosis, tubular apoptosis↑ tubular proliferation	[44]
UUO in rat	Mitochondrial protection, antioxidative, antiapoptotic	↓Collagen I, fibronectin, *α*-SMA, mtROS, caspase 3,7,9	NA	↓Renal fibrosis, tubular apoptosis	[59]
Uninephrectomy and STZ-induced CD1 mice	Antioxidative Antiapoptotic	↓Nox4, TXNIP, TGF-*β*, fibronectin, collagen IV, Bax, p38 MAPK, CREB ↑Bcl-2	↓Proteinuria, urinary 8-OHdG	↓Glomerular hypertrophy, mesangial expansion	[15]
STZ-induced C57BL/6 mice	Mitochondrial protection	↓Drp1, Bax, caspase 1, IL-1*β* ↑Mfn1, MDA ↑SOD, GSH-PX, Bcl-2,	↓Proteinuria, Scr, BUN	↓Tubulointerstitial fibrosis, mesangial matrix proliferation	[60]
db/db mouse	Mitochondrial protection	↓Pla2 ↑LCLAT1, Mfn1	↓Albuminuria, urinaryH2O2, urinary ACR	↓Mesangial matrix accumulation	[52]
db/db mice	Antioxidative, antilipid deposition	↑ MnSOD, CAT ↓NADPH oxidase, CD36, fibronectin, collagen IV, NF-*κ*B	↓Proteinuria, Scr, urinary 8-OHdG, urinary MDA	↓Glomerular hypertrophy, tubular injury	[42]
BTBR^ob/ob^ and Abca1^fl/fl^ ob/ob mice	Mitochondrial protection	NA	↓ACR, BUN, Scr, albuminuria	↑Podocyte number ↓mesangial expansion	[51]
HFD-induced C57BL/6	Mitochondrial protection, antiapoptotic, anti-inflammatory	↓TNF-*α*, MCP-1, NF-*κ*B, VEGF, TGF-*β*↑p-AMPK	NA	↓Glomerulosclerosis, mesangial expansion ↑podocytes, endothelial cells	[23]
Diet-induced MetS in pig	Mitochondrial protection	↑PPAR-*α*, P62, Bcl-xl ↓caspase 3	Scr, GFR (no effect)	↓Medullary volume, medullary hypoxia	[61]

∗IR: ischemia-reperfusion; AKI: acute kidney injury; STZ: streptozocin; Scr: serum creatinine; Ccr: creatinine clearance; BUN: blood urea nitrogen; GFR: glomerular filtration rate; GSH: glutathione; MDA: malondialdehyde; HO-1: heme oxygenase-1; iNOS: nitric oxide synthase; MPO: myeloperoxidase; TNF-*α*: tumor necrosis factor-alpha; NF-*κ*B: nuclear factor kappa B; TGF-*β*: transforming growth factor-*β*; SOD: superoxide dismutase; ROS: reactive oxygen species; FeNa%: fractional excretion of sodium; FeK%: fractional excretion of potassium; MCP-1: monocyte chemoattractant protein; PGC*α*: peroxisome. Proliferator–activated receptor-*γ*-coactivator; PPAR-*α*: peroxisome proliferator–activated receptor; VEGF: vascular endothelial growth factor; UUO: unilateral ureteral obstruction; mtROS: mitochondrial ROS; TXNIP: thioredoxin-interacting protein; 8-OHdG: 8-hydroxy-2-deoxyguanosine; ACR: albumin to creatinine ratio; Pla2: phospholipase A2; LCLAT1: lysocardiolipin acyltransferase 1; MnSOD: Mn superoxide dismutase; CAT: catalase; HFD: high-fat diet; MetS: metabolic syndrome; NA: not described.

**Table 2:** In vitro study of SS-31 in kidney disease.

Cell type	Protective effect	Cytokines	References
Swine renal artery endothelial cells incubated with tert-butyl hydroperoxide	Mitochondrial protection	↓Caspase-3 ↑eNOS	[49]
HG-induced mouse mesangial cells	Mitochondrial protection, antiapoptotic, antioxidative	↓Bax/Bcl-2, cleaved caspase-3, TGF-*β*, fibronectin, ROS, TXNIP, p38 MAPK ↑TRX	[15]
HG-human proximal tubular epithelial cells incubation with SS-31 or Drp1 inhibitor	Mitochondrial protection	↓mtROS, Drp1, caspase1, IL-1*β* ↑MMP, Mfn1	[60]
HG-human proximal tubular epithelial cells co-incubated with SS-31	Antioxidative, antilipid deposition	↑ MnSOD, CAT ↓NADPH oxidase, Nox4, CD36, NF-*κ*B	[42]
Human proximal tubular epithelial cells incubated with cisplatin or combined with SS-31	Antioxidative, antiapoptotic	↓mtROS, NLRP3, IL-1*β*, caspase 1	[37]

∗HG, 30 mM glucose; ROS: reactive oxygen species; mtROS: mitochondrial ROS; MMP: mitochondrial membrane potential; TXNIP: thioredoxin-interacting protein; TGF-*β*: transforming growth factor-*β*; MnSOD: Mn superoxide dismutase; CAT: catalase; NF-*κ*B: nuclear factor kappa B.
